# A highly soluble, non-phototoxic, non-fluorescent blebbistatin derivative

**DOI:** 10.1038/srep26141

**Published:** 2016-05-31

**Authors:** Boglárka H. Várkuti, Miklós Képiró, István Ádám Horváth, László Végner, Szilvia Ráti, Áron Zsigmond, György Hegyi, Zsolt Lenkei, Máté Varga, András Málnási-Csizmadia

**Affiliations:** 1 Department of Biochemistry, Institute of Biology, Eötvös Loránd University, H-1117 Budapest, Hungary; 2 Printnet Ltd., H-2212 Veresegyház, Hungary; 3 Department of Genetics, Institute of Biology, Eötvös Loránd University, H-1117 Budapest, Hungary; 4 Brain Plasticity Unit, ESPCI-Paris-Tech, CNRS UMR8249, Paris, France; 5 MTA-ELTE Molecular Biophysics Research Group, Department of Biochemistry, Eötvös Loránd University, H-1117 Budapest, Hungary; 6 Optopharma Ltd., H-1015 Budapest, Hungary

## Abstract

Blebbistatin is a commonly used molecular tool for the specific inhibition of various myosin II isoforms both *in vitro* and *in vivo*. Despite its popularity, the use of blebbistatin is hindered by its poor water-solubility (below 10 micromolar in aqueous buffer) and blue-light sensitivity, resulting in the photoconversion of the molecule, causing severe cellular phototoxicity in addition to its cytotoxicity. Furthermore, blebbistatin forms insoluble aggregates in water-based media above 10 micromolar with extremely high fluorescence and also high adherence to different types of surfaces, which biases its experimental usage. Here, we report a highly soluble (440 micromolar in aqueous buffer), non-fluorescent and photostable C15 amino-substituted derivative of blebbistatin, called para-aminoblebbistatin. Importantly, it is neither photo- nor cytotoxic, as demonstrated on HeLa cells and zebrafish embryos. Additionally, para-aminoblebbistatin bears similar myosin II inhibitory properties to blebbistatin or para-nitroblebbistatin (not to be confused with the C7 substituted nitroblebbistatin), tested on rabbit skeletal muscle myosin S1 and on M2 and HeLa cells. Due to its drastically improved solubility and photochemical feature, as well as lack of photo- or cytotoxicity, para-aminoblebbistatin may become a feasible replacement for blebbistatin, especially at applications when high concentrations of the inhibitor or blue light irradiation is required.

Blebbistatin^1^ is a widely used and well-characterized small molecular inhibitor of class II myosins, a group of ATP driven molecular motors acting in concert with actin to form a contracting actomyosin network. Myosin IIs are responsible for various cellular and physiological functions such as muscle contraction, cytokinesis, differentiation, polarization, cell motility and blebbing^2^,^3^. Since blebbistatin blocks myosin heads in a low actin-affinity state, it prevents the formation of strongly-bound non-functional actomyosin complexes, which make blebbistatin a useful tool in the study of muscle physiology and cellular actomyosin networks^4^,^5^.

Despite its popularity, blebbistatin has several disadvantageous chemical properties, such as photoinstability, cyto- and phototoxicity, high fluorescence and very poor water solubility as above 10 μM concentration its slow precipitation can be observed^6^,^7^,^8^,^9^. Since the myosin inhibitory constants of blebbistatin are in a similar range as its solubility, this property may significantly affect the apparent experimental results. Additionally, the high fluorescence of blebbistatin and poor water solubility results in fluorescent aggregates which can become serious obstacles in light scattering or GFP-based experiments or may damage the vascular system of model animals^10^,^11^. Furthermore, due to the blue light sensitivity of blebbistatin, irradiation below 500 nm results in the photoconversion of the molecule, accompanied by the generation of reactive oxygen species responsible for the phototoxic effect^6^,^7^,^9^. As a result, irradiation or imaging human cells or animal model systems below 500 nm in the presence of blebbistatin severely damages the samples^8^,^9^,^12^,^13^. Blebbistatin exerts cytotoxicity even without irradiation in long-term experiments^8^,^9^. Therefore, a non-fluorescent, photostable, non-cytotoxic and non-phototoxic derivative of blebbistatin has been developed recently, named para-nitroblebbistatin, which contains an electron-withdrawing nitro group at the C15 (para) position^8^. We note that para-nitroblebbistatin is a highly specific inhibitor in contrast with the C7 nitro substituted blebbistatin derivative (commonly called nitroblebbistatin) which exerts relatively low affinity to myosin II (IC_50_ = 27.5 μM). Importantly, the *in vitro* and *in vivo* myosin II inhibitory properties of para-nitroblebbistatin are not affected by the nitro substitution. Despite its highly advantageous properties over blebbistatin, the poor water solubility of para-nitroblebbistatin, similarly to blebbistatin, may still restrict its application.

Blebbistatin inhibits the actin-activated ATPase activity of rabbit skeletal, porcine cardiac and scallop striated myosins, vertebrate non-muscle myosin lls and *Dictyostelium discoideum* myosin II with an IC_50_ of 0.5–5 μM^14^. On the other hand, the inhibition of the actin activated ATPase activity of smooth muscle and *Acanthamoeba* myosin II requires much higher blebbistatin concentrations (IC_50_ of 79.6 and 83 μM, respectively). Regardless of IC_50_ values and blebbistatin’s limited solubility (from 7 to 20 μM, depending on the conditions of the measurements)^8^,^15^, the inhibitor is usually applied at the concentration of 50–100 μM. At such high concentrations, slow precipitation of blebbistatin occurs at the time-scale of hours. Therefore, the application of blebbistatin at higher concentrations than its solubility faces several hurdles in long-term experiments: I) the concentration of the inhibitor decreases over time, and so does its inhibitory effect, II) the light scattering of the media gradually increases, confounding/perturbing light-scattering based measurements, III) the precipitated aggregates have high fluorescence hampering imaging, IV) the aggregates can block the vascular system of animals in *in vivo* studies^10^. Furthermore, since the aggregates re-dissolve very slowly and have the tendency to attach to different surfaces^10^, complete blebbistatin wash-out from *in vitro* and *in vivo* samples is rather problematic. Such features resulting from the low solubility of blebbistatin in aqueous media hinders its general usage and biases its reversibility in many experimental setups.

The electron withdrawing nitro substitution at the C15 position diminishes blebbistatin’s cyto- and phototoxicity, reduces its fluorescence and increases its photostability^8^. Based on these observations we speculated that substituting a polar, electron withdrawing group at this position may not only offer the benefits of para-nitroblebbistatin but would also elevate the water solubility of the new derivative. Based on this assumption, we synthesized para-aminoblebbistatin, whose protonated amino group at physiological pH provides the desired positive charge and a strong electron withdrawing characteristic. We also present the *in vitro* and *in vivo* myosin II inhibitory features of para-aminoblebbistatin on several different myosin isoforms. Importantly, we demonstrate that the new derivative of blebbistatin is non-fluorescent, photostable, non-cytotoxic, non-phototoxic while its solubility is more than 40x higher than blebbistatin’s or para-nitroblebbistatin’s. Para-aminoblebbistatin forms a stable solution in aqueous buffers and does not precipitate.

## Results

### Synthesis of para-aminoblebbistatin

**Figure 1:**
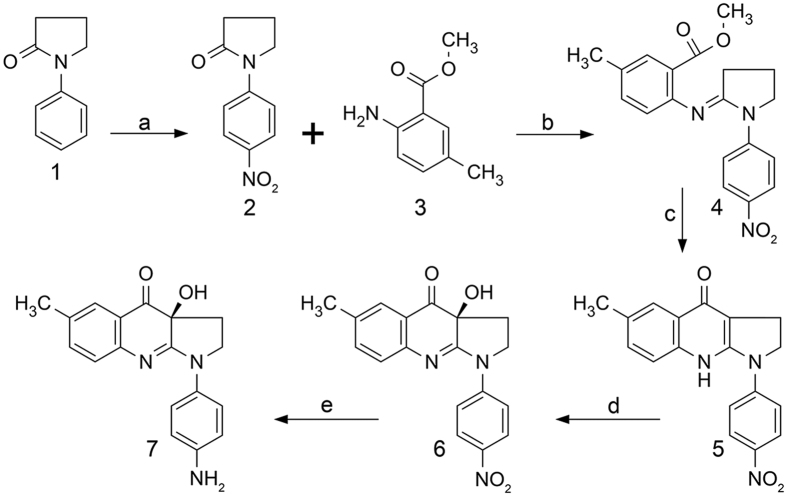
Synthesis of para-aminoblebbistatin. Reagents and conditions: (**a**) H_2_SO_4_, HNO_3_, 0 °C, 15 min; (**b**) POCl_3_, CH_2_Cl_2_, 50 °C, 18 hours; (**c**) LiHMDS, −78 °C to 0 °C, 3 hours; (**d**) oxaziridine, −10 °C, 16 hours; (**e**) NH_4_HCO_2_, Pd black, CH_3_OH, RT, 18 hours.

We have demonstrated that the C15 position of blebbistatin can be modified without affecting its myosin II inhibitory properties^8^,^15^. Electron withdrawing substitutions at this position – such as chloro or nitro groups – not only quench the fluorescence of blebbistatin but also elevate its photostability. Furthermore, C15 nitro substitution eliminates both the blue light phototoxicity and the cytotoxicity of blebbistatin. In order to get a photostable, non-fluorescent and a highly soluble blebbistatin derivative we synthesized its C15 amino-substituted form. Para-aminoblebbistatin was synthesized by the reduction of para-nitroblebbistatin in the presence of ammonium formate using palladium black catalyst (Fig. 1). Para-nitroblebbistatin was synthesized according to published protocols^8^.

### Physico-chemical characterization of para-aminoblebbistatin

**Figure 2:**
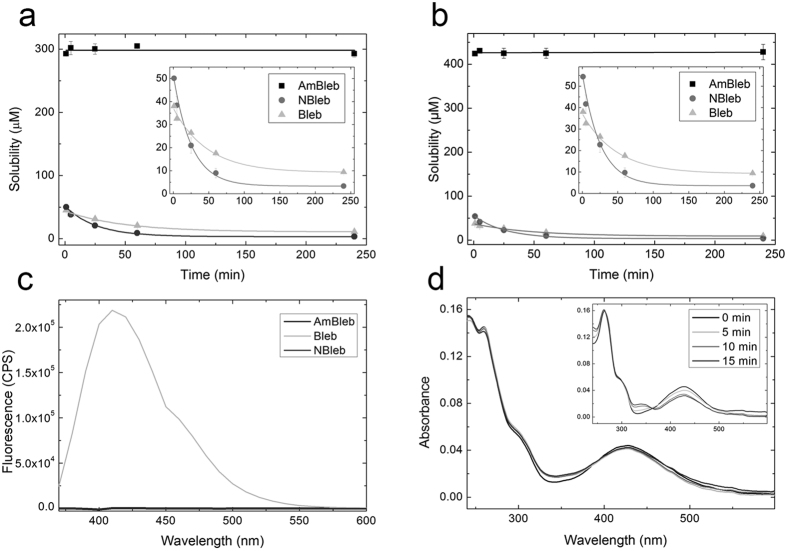
Physico-chemical properties of para-aminoblebbistatin (AmBleb), para-nitroblebistatin (NBleb) and blebbistatin (Bleb). (**a**) Solubility of AmBleb, NBleb and Bleb in 0.1 vol/vol% DMSO in assay buffer in time. After the centrifugation of a 500 μM of AmBleb suspension in assay buffer yielded 298 ± 2.5 μM soluble supernatant concentration. The concentration of this solution stayed constant for 4 hours. Supernatant concentrations of 50 μM of NBleb and Bleb decreased exponentially after centrifugation at different lengths of time (enlarged in the inset), reaching equilibria at 3.3 ± 0.1 μM and 10.9 ± 0.9 μM, respectively (obtained from fitting the data to single exponential functions). (**b**) Solubility of AmBleb, NBleb and Bleb in 1 vol/vol% DMSO are 426 ± 1.7 μM, 3.6 ± 0.2 μM and 9.3 ± 0.7 μM, respectively (enlarged in the inset, obtained from the exponential fits to data). (**c**) Fluorescence spectra of AmBleb, NBleb and Bleb at λ_exc_ = 350 nm. (**d**) Absorbance spectra of AmBleb and Bleb (inset) after irradiating 5 μM of the inhibitors at 480 ± 10 nm for different lengths of time.

We measured the solubility and solution stability of para-aminoblebbistatin, para-nitroblebbistatin and blebbistatin in 0.1 and 1 vol/vol% DMSO at room temperature. 50 μM of the inhibitors were dissolved in assay buffer (see Experimental Procedures) containing 0.1 or 1 vol/vol% DMSO, centrifuged at the indicated times and the concentration of the supernatants were determined at each time point (Fig. 2a,b). In two hours blebbistatin and para-nitroblebbistatin solutions reached equilibrium, yielding solubility values of 10.9 ± 0.9 μM and 3.3 ± 0.1 μM in 0.1 vol/vol% DMSO and 9.3 ± 0.7 μM and 3.6 ± 0.2 μM in 1 vol/vol% DMSO, respectively (enlarged in the insets of Fig. 2a,b). 50 μM para-aminoblebbistatin stayed stable in solution during the whole experiment in both 0.1 and 1 vol/vol% DMSO. Tha saturation concentrations for para-aminoblebbistatin were determined as 298 ± 2.5 μM and 426 ± 1.7 μM in 0.1 vol/vol% DMSO and 1 vol/vol% DMSO respectively (Fig. 2a,b). At these concentrations, the solutions were stable even for several days.

Blebbistatin has high fluorescence with an emission peak at 410 nm, which can be a serious disadvantage in fluorescence-based experiments. In contrast, para-aminoblebbistatin, similarly to para-nitroblebbistatin, is non-fluorescent due to the electron withdrawing substitution at the C15 position (Fig. 2c). Besides its high fluorescence blebbistatin is also photodegradable and can be photo-inactivated, which is accompanied by the alteration of its absorbance spectra reflecting changes in its molecular structure^6^,^9^,^16^. We compared para-aminoblebbistatin’s photostability to blebbistatin’s by irradiating the molecules in assay buffer at 480 ± 10 nm for different lengths of time (which is a typical GFP excitation range) and recorded the absorption spectra afterwards (Fig. 2d). The spectra of blebbistatin changed markedly indicating photo-induced changes in the molecular structure, consistent with previously published results (inset of Fig. 2d). In contrast, absorption spectrum of the para-aminoblebbistatin solution have not altered considerably, demonstrating significantly improved photostability, similarly to para-nitroblebbistatin^8^.

### *In vitro* and *in vi*vo inhibitory properties of para-aminoblebbistatin

**Figure 3:**
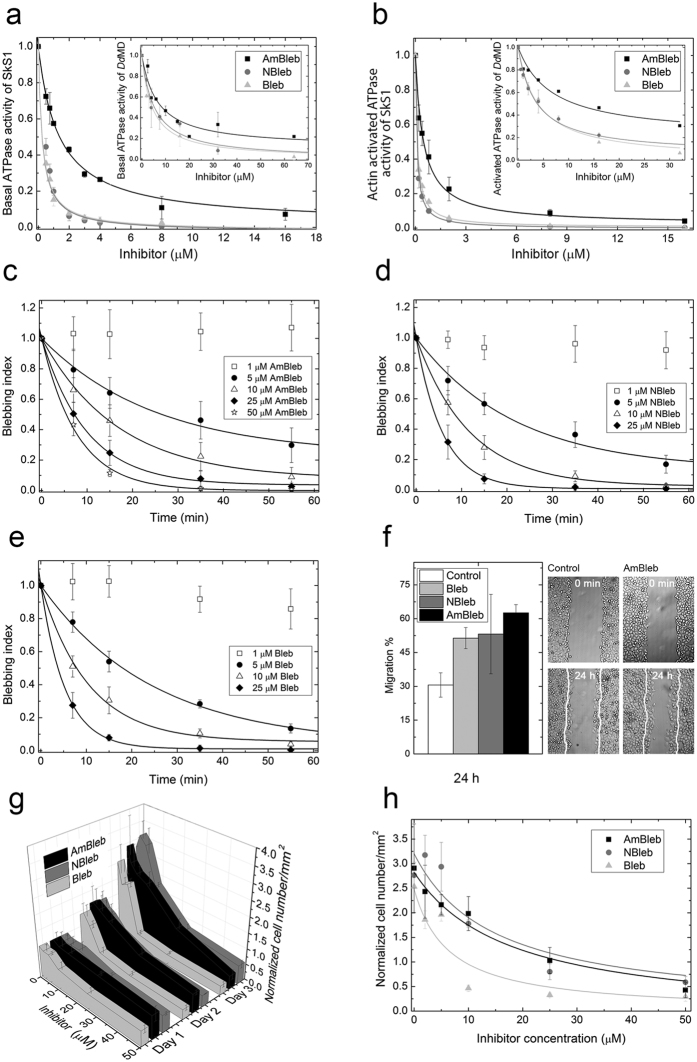
*In vitro* and *in vivo* inhibitory properties of para-aminoblebbistatin, para-nitroblebbistatin and blebbistatin. *In vitro* inhibition of basal (**a**) and actin activated (**b**) ATPase activity of rabbit skeletal S1 (SkS1) and *Dictyostelium discoideum* myosin II motor domain (*Dd*MD) at increasing concentrations of AmBleb, NBleb and Bleb. Data (means ± s.d. from three independent experiments) were normalized to ATPase activities at 0 μM inhibitor concentrations and were fitted with hyperbolic functions. (**c–e**) Blebbing indices of M2 cells in the presence of increasing concentrations of AmBleb (**c**), NBleb (**d**) and Bleb (**e**) monitored for 60 minutes. Blebbing indices are means and standard deviations/means and s.d./means ± s.d. (n = 6) of the number of blebs formed in a 5-minute interval on a cell^17^. Blebbing index values are normalized to the starting point. Data were fitted with exponentials yielding rate constants of inhibition for 5, 10, 25 and 50 μM of the inhibitors (see Table 1.) (**f**) Relative migration of HeLa cells measured after 24 h in the absence (Control) or in the presence of 20 μM AmBleb, NBleb and Bleb (left panel). 100% is considered as the total diameter of the wound (n = 6 scratches, from two independent experiments). Representative images of wound healing assays immediately after scratching (0 min) and after 24 h incubation with AmBleb or without the inhibitors (Control). White lines mark the cell edges at 0 minutes (**g**). Inhibition of cell number growth of HeLa cells by increasing concentrations of AmBleb, NBleb and Bleb monitored for three days. Cell numbers were normalized to 0 μM inhibitor concentrations counted on the first day of the experiment. (**h**) Cell number as a function of increasing inhibitor concentrations at day 3 of the experiment presented in (**g**). Data (means ± s.d., n = 50–100) were fitted with hyperboles.

After the physico-chemical characterization of para-aminoblebbistatin, we compared its *in vitro* and *in vivo* inhibitory properties to those of blebbistatin and para-nitroblebbistatin. We measured the basal and actin activated ATPase activites of rabbit skeletal muscle myosin S1 (SkS1) and *Dictyostelium discoideum* myosin II motor domain (*Dd*MD) at increasing concentrations of para-aminoblebbistatin, para-nitroblebbistatin or blebbistatin (Fig. 3a,b). All three myosin II inhibitors decreased the basal as well as actin activated ATPase activity of SkS1 to a maximal extent (~98–100%) yielding half-maximal inhibitory concentration values of IC_50,AmBleb_ = 1.3 ± 0.1 μM, IC_50,NBleb_ = 0.3 ± 0.04 μM and IC_50,Bleb_ = 0.3 ± 0.03 μM for the basal ATPase of SkS1 and IC_50,AmBleb_ = 0.47 ± 0.06 μM, IC_50,NBleb_ = 0.1 ± 0.004 μM and IC_50,Bleb_ = 0.11 ± 0.009 μM for the actin activated ATPase activity of SkS1. Para-nitroblebbistatin and blebbistatin also exerted maximal inhibition on *Dd*MD (~100%), while AmBleb reached 90% and 80% inhibition on basal and actin activated ATPase activities of *Dd*MD yielding half-maximal inhibitory concentration values of IC_50,AmBleb_ = 6.6 ± 2 μM, IC_50,NBleb_ = 5.3 ± 1.6 μM and IC_50,Bleb_ = 4.4 ± 0.3 μM for the basal ATPase and IC_50,AmBleb_ = 6.7 ± 1.9 μM, IC_50,NBleb_ = 3.4 ± 0.3 μM and IC_50,Bleb_ = 3.9 ± 0.3 μM for the actin activated ATPase activity of *Dd*MD. It should be noted that IC_50_ of AmBleb is higher by 4.3 and 1.7 times on SkS1 and *Dd*MD, respectively.

Next, we compared the non-muscle myosin II inhibition by para-aminoblebbistatin, para-nitroblebbistatin and blebbistatin in human melanoma (M2) and HeLa cells. As blebbistatin acquired its name from its ability to block blebbing of M2 cells^1^, we compared blebbing indices^17^ of M2 cells in time, incubated in the three different myosin II inhibitors at different concentrations (Fig. 3c–e). Even 5 μM concentrations of the myosin II inhibitors achieved significant decrease in the blebbing indices of the cells within an hour, whereas higher concentrations of the inhibitors completely stopped blebbing of the cells within >40–60 minutes (10 μM) or >20 minutes (25 and 50 μM). The rate constants for the inhibition of blebbing of M2 cells at 5 μM inhibitor concentrations were similar, whereas at 10 and 25 μM concentration of para-aminoblebbistatin the rate constants were slightly slower than those of para-nitroblebbistatin or blebbistatin (Table 1).

**Figure 4:**
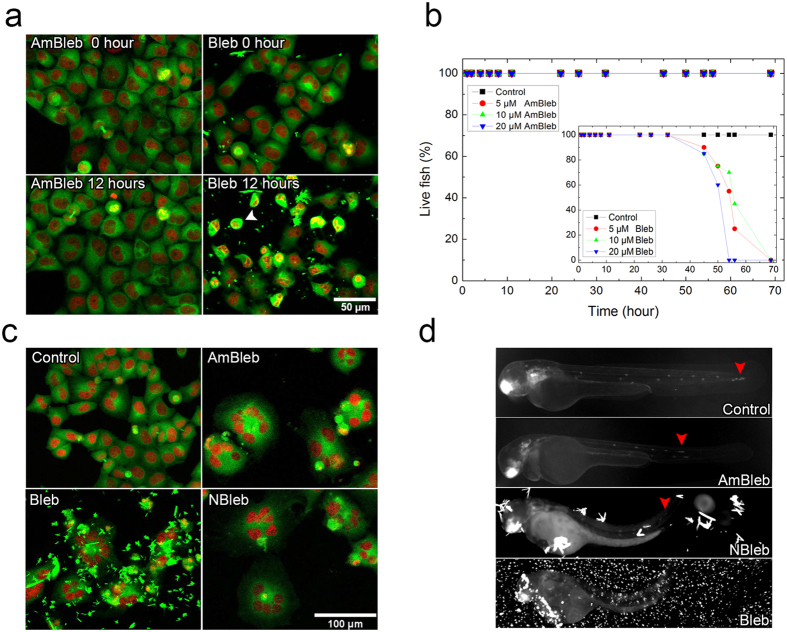
Phototoxicity and cytotoxicity assays with para-aminoblebbistatin or blebbistatin and fluorescent imaging in the presence of the myosin II specific inhibitors. (**a**) Maximum intensity projections of fluorescent confocal microscopic z-stack images of EGFP-α-tubulin H2B-mCherry HeLa Kyoto cells at the beginning and at the end of a 12-hour time-lapse imaging in the presence of 50 μM AmBleb or Bleb. Blebbistatin treated cells display severe phototoxicity (white arrowhead). (**b**) Lifespan cytotoxicity assays of zebrafish embryos (n = 20) incubated at increasing concentrations of AmBleb and Bleb (inset) in dark. (**c**) Fluorescent confocal microscopic images of HeLa Kyoto cells in the presence of 50 μM inhibitor concentrations on the 3^rd^ day of the experiment presented in Fig. 3e,f. (**d**) 48 hpf (hours-post-fertilization) *cldnb:EGFP* zebrafish embryos in the absence (Control) and in the presence of 20 μM of the myosin II inhibitors after 24 hours of incubation. Red arrowheads mark the position of the pLLps.

Blebbistatin also inhibits or enhances motility of different cell lines on different substrates and on 2D or 3D environments^18^,^19^, and seems to moderately enhance the motility of HeLa cells on 2D surfaces (~140% compared to 100% of the control)^19^. To test the effect of the different myosin II inhibitors on the motility of HeLa cells on uncoated glass surface, we carried out wound-healing assays in the absence and in the presence of 20 μM concentrations of the inhibitors (Fig. 4f). All three inhibitors increased motility of HeLa cells compared to untreated cells, quantified after 24 hours of incubation (30.6 ± 5.4% for control, 62.6 ± 3.6% for AmBleb, 53.2 ± 17.6% for NBleb and 51.4 ± 4.7% for Bleb, where 100% is considered as the total diameter of the wound).

Another important feature of blebbistatin and para-nitroblebbistatin is that they block cytokinesis and induce multinucleated cells through the inhibition of non-muscle myosin II, thereby keeping low cell number in human cancer cell cultures^1^. Therefore, we followed cell number of HeLa cells in time at different inhibitor concentrations of para-aminoblebbistatin and compared the results to those of para-nitroblebbistatin- and blebbistatin-treated cells (Fig. 3g). Cells incubated in different concentrations of the inhibitors were counted on each day for three days. On the 3^rd^ day of the experiment, cell number in the absence of inhibitors (0 μM) increased to 3–3.5 times of the initial cell number, whereas 25 or 50 μM of the inhibitors completely inhibited cytokinesis resulting in no significant increase in cell number. The IC_50_ values of the inhibition of cell proliferation (Fig. 3h) were similar in the case of para-aminoblebbistatin and para-nitroblebbistatin (IC_50,AmB_ = 17.8 ± 4.7 μM, IC_50,NB_ = 14.7 ± 4.2 μM) but 2–3 times weaker than blebbistatin’s (IC_50,B_ = 6.4 ± 4.8 μM).

Since the myosin II inhibitors decrease the ATPase activity of SkS1 *in vitro*, we tested their inhibition on zebrafish skeletal muscle *in vivo*. We investigated the fast escape response (C-start) reflex of 6 dpf fish evoked by standardized tapping stimuli (Video 1). First, the head of the zebrafish embryo rotates about the center of mass towards the direction of future escape, then the body exhibits a curvature that resembles a letter C, mediated by quick contraction of the muscles on one side accompanied by relaxation on the other side^20^. The velocity of the rotating heads of the embryos were quantified (maximal angular velocity) in the absence and in the presence of increasing concentrations of the myosin II inhibitors (2, 5, 10, 20 μM), monitored for 4 hours. The half maximal effective concentration (EC_50_) values for the inhibition of the maximal angular velocity by the myosin II inhibitors are below 2 μM since even at 2 μM the inhibitory effects were >90% for all inhibitors. We experienced that the inhibitory effect of para-aminoblebbistatin is exerted much slower than that of blebbistatin and para-nitroblebbistatin, probably due to its slower uptake (half inhibitions at 0.6, 1.3 and 3.2 hours, respectively) (Supplementary Figure 1). To further evaluate the specificity of blebbistatin and its derivatives on different myosin II isoforms, we examined the effect of the inhibitors on 6 dpf (days post fertilization) zebrafish heart muscle *in vivo*. We monitored heart beating and the amplitude of heart muscle contraction before and after the addition of 20 μM of the inhibitors to the specimens. By the addition of the inhibitors the pulse rate (heart beats/time) did not change substantially in the first three hours (between 120 and 130 beats/minute). In contrast, the amplitude of contraction and consequently the blood circulation dropped drastically within the first hour in blebbistatin and para-nitroblebbistatin and within three hours in para-aminoblebbistatin (Video 2).

### Enhanced imaging by the non-phototoxic and non-cytotoxic para-aminoblebbistatin

The blue light phototoxicity of blebbistatin is one of its most annoying limitations, which makes live-cell fluorescence microscopy unfeasible applying blue light excitations. To test the applicability of para-aminoblebbistatin in live-cell fluorescence microscopy, we time-lapse imaged EGFP-α-tubulin H2B-mCherry labeled HeLa Kyoto cells in a confocal microscope (Fig. 4a). Cells were treated with 50 μM para-aminoblebbistatin or blebbistatin and imaged for 12 hours applying 3 z-sections every 10 minutes. Cells receiving para-aminoblebbistatin treatment did not show any sign of phototoxicity, while numerous blebbistatin-treated cells rounded up from the surface and eventually died within a few hours of imaging. This was accompanied by their increased autofluorescence and fragmented, dense nuclei (increased green fluorescence of cytoplasm and red fluorescence of nuclei, co-localizing as yellow, an example marked by a white arrowhead in Fig. 4a). Additionally, microscopic images of blebbistatin-treated cells were highly disturbed by the fluorescent precipitates of blebbistatin formed at this concentration.

In live zebrafish embryo tests, myosin II inhibition by blebbistatin proved to be highly toxic after 24 hours of incubation, whereas using para-nitroblebbistatin the cytotoxic effect was negligible^8^. In order to compare the cyototoxicity of para-aminoblebbistatin to that of blebbistatin, we treated zebrafish embryos with the inhibitors at different concentrations and followed their development for 72 hours and determined their lifespan (Fig. 4b). After 40 hours, blebbistatin-treated zebrafish embryos started to die, and at 69 hours all of them died at all blebbistatin concentrations. In contrast, embryos treated with para-aminoblebbistatin did not show any sign of cytotoxicity even at high concentrations and their fitness was comparable to the untreated control embryos.

Multinuclearity of the inhibitor-treated HeLa cells can be demonstrated well on the 3^rd^ day of incubation in the presence of 50 μM para-aminoblebbistatin, para-nitroblebbistatin or blebbistatin from the experiment presented in Fig. 3e,f (Fig. 4c). However, the highly fluorescent, dense precipitates of blebbistatin disturb imaging in the GFP channel of the microscope. Since para-aminoblebbistatin is soluble at this concentration, it does not form aggregates. Although para-nitroblebbistatin is not soluble at 50 μM concentration, the aggregates are not fluorescent using 488 nm excitation by confocal micrsocopy, thus they do not affect imaging. Interestingly, using 470 ± 20 nm excitation by an epifluorescent microscope, the aggregates of para-nitroblebbistatin are visible and also disturb imaging, while blebbistatin aggregates in this case completely hindered the observation of the GFP signal. In this experiment, *cldnb:gfp* zebrafish embryos were imaged by an epifluorescent microscope after 24 hours of incubation in the presence of 20 μM concentrations of the indicated inhibitors (Fig. 4d) in order to detect the effect of myosin II inhibition on the proliferating leading edge of the posterior lateral line primordium (pLLp)^21^ and visualize the effect of the precipitates by epifluorescent microcopy. Similarly to para-nitroblebbistatin and blebbistatin, para-aminoblebbistatin also halted pLLp migration, indicating *in vivo* myosin II inhibition^4^,^8^,^22^. In the case of para-aminoblebbistatin treatment, the GFP signal of the migrating pLLp can be visualized clearly, while the precipitates of the two other inhibitors highly disturbed the imaging.

## Discussion

Blebbistatin is the most commonly used myosin II inhibitor, but its applicability is greatly hampered by its low solubility as well its sensitivity to blue light. In the majority of the published studies, blebbistatin is applied at 50–100 μM concentrations, although at such concentrations blebbistatin precipitates and the effective concentration exponentially drops to the equilibrium saturation concentration (9 μM) in two hours. Since the precipitated aggregates have the propensity of attaching to surfaces like the wall of the Eppendorf tube or the pipette, the actual concentration of the inhibitor becomes uncertain during the experimental procedures. This effect significantly decreases the reproducibility of the studies and causes serious problems for researchers. Furthermore, the solubility saturation concentration of blebbistatin is often in the similar range as the EC_50_ of its myosin II inhibition, consequently, most of these myosin II molecules cannot be saturated with blebbistatin in aqueous buffers.

According to our experience and other published studies, increasing blebbistatin concentrations to higher values than its solubility was accompanied by an increased inhibitory effect as well. In order to resolve this discrepancy, we followed the precipitation kinetics of blebbistatin and found that its initial apparent solubility in fact can be much higher than its saturation concentration. Thus, a freshly prepared blebbistatin sample can reach even 50 μM before it drops to its half concentration in <20 minutes. Further complications may arise from the uncertainty of blebbistatin’s actual concentrations in membranes and within cells. Additionally, low actual concentrations cause significant time delays in myosin II inhibition after blebbistatin addition both *in vitro* and *in vivo*. As an example, the on-rate constant of blebbistatin for binding to rabbit skeletal muscle myosin II is relatively low (k_on_ = 0.0034 μM^−1^ s^−1^)^5^. Therefore, it requires more than a minute for 10 μM of blebbistatin to bind to 90% of rabbit skeletal myosin, even though this myosin isoform is among the strongest blebbistatin binders within myosin IIs. Thus, for a more rapid inhibition, much higher blebbistatin concentrations may be applied. Further difficulties may rise during *in vitro* light scattering or fluorescent spectroscopic measurements as well due to the precipitation of blebbistatin.

All issues described above can be overcome by the use of para-aminoblebbistatin, because it is highly soluble and forms stable solutions even at over 100 μM concentrations. Additionally, it is non-fluorescent both in its unbound form and in complex with proteins. Para-aminoblebbistatin is specific to skeletal muscle myosin *in vitro* and *in vivo*, heart muscle myosin *in vivo*, Dictyostelium myosin II and non-muscle myosin IIs, although its binding constants are slightly lower compared to blebbistatin or para-nitroblebbistatin. Further important advantages of para-aminoblebbistatin over blebbistatin are its low phototoxicity and cytotoxicity, which are similar to those of para-nitroblebbistatin. Additionally, both para-aminoblebbistatin and para-nitroblebbistatin are highly photostable, which may be an important feature in different experimental setups where continuous blue-light irradiation is applied. Since blebbistatin degrades relatively rapidly under blue light, its activity decreases quickly biasing its actual concentration.

In summary, para-aminoblebbistatin is a useful replacement of blebbistatin in myosin II inhibition experiments. Para-aminoblebbistatin provides a great advantage when high concentration of the inhibitor is required, cytotoxic or phototoxic effects cause difficulties or fluorescence imaging would be disturbed by blebbistatin.

## Methods

### Synthesis of para-aminoblebbistatin

3.6 mg para-nitroblebbistatin (synthesized as in^8^) was dissolved in 0.7 ml methanol and it was mixed with 0.7 ml cc. ammonium formate in methanol solution. A few granules of palladium black were added and the mixture was stirred for 18 hours at room temperature. A Strata XL 2g SPA column (Phenomenex) was equilibrated with 50 ml acetonitrile, then 50 ml water and the crude reaction mixture was pipetted onto the column. It was sequentially washed with 20 ml water, then eluted with 20 ml acetonitrile containing 1% trifluoroacetic acid and dried in vacuum. The brownish oil was dissolved in 0.5 ml acetonitril containing 0.1% triethylamine (TEA). The product was isolated by HPLC (Agilent 1100 instrument) using Luna 250 × 10 mm C18(2) column (Phenomonex). The HPLC conditions were as follows: isocratic elution, content of buffer: water (0.1% TEA): acetonitrile (0.1% TEA) = 3:7, flow rate: 3, 5 ml/min, detection wavelength: 254 nm. Ms (ESI): *m*/*z* = 308.1 [M + H]^+^. Chemical purity: consistent with >95% purity (LC, 210 nm). Appearance: orange solid.

### Solubility assays

500 μM para-aminoblebbistatin and 50–50 μM para-nitroblebbistatin and blebbistatin solutions were prepared in assay buffer (40 mM NaCl, 4 mM MgCl_2_, 20 mM HEPES pH 7.3). The DMSO content was 1%. After 0, 5, 25, 60 and 240 minutes, the samples were centrifuged at 13,000 g for 1 minute. Absorption spectra of the supernatants were recorded and the concentrations of the dissolved inhibitors were determined using the following extinction coefficients: para-aminoblebbistatin (ε°_427_ = 6860 M^−1^cm^−1^), blebbistatin (ε°_427_ = 6100 M^−1^cm^−1^), para-nitroblebbistatin (ε°_427_ = 11175 M^−1^cm^−1^). The experiments were repeated three times.

### Conditions of spectral measurements

Fluorescence emission spectra (λ_exc_ = 350 nm) were recorded in assay buffer containing 0.1% DMSO applying the inhibitors at 5 μM concentrations. The photoconversion measurements were carried out using 5 μM para-aminoblebbistatin or blebbistatin in assay buffer and irradiating the samples at 480 ± 10 nm for 0, 5, 10 and 15 minutes using a Fluorescence Spectrometer (F900, Edinburgh Instruments) equipped with a 450 W Xenon Lamp. Their absorbance spectra were measured at each time point.

### Steady state ATPase measurements

MgATPase activities of 500 nM (for basal ATPase) or 50 nM (for actin activated ATPase) SkS1 (rabbit skeletal S1 fragment)^23^ and 1 μM (for basal ATPase) or 300 nM (for actin activated ATPase) *Dd*MD were measured at increasing concentrations of blebbistatin, para-nitroblebbistatin or para-aminoblebbistatin in the absence or in the presence of 45 μM actin for SkS1 or 15 μM actin for *Dd*MD. Measurements were carried out using a pyruvate kinase/lactate dehydrogenase coupled assay (NADH-coupled assay) [5] at 25 °C or 20 °C in case of SkS1 or *Dd*MD, respectively. Myosin and actin together with the indicated concentration of the inhibitor were pre-incubated for 5 minutes at room temperature and the measurements were initiated with 1 mM ATP. Data were corrected for background ATPase activity of actin. G-actin was prepared accordingly from rabbit skeletal muscle^24^ and polymerized by 2 mM MgCl_2_ for 1 hour at room temperature. Measurements were performed in low salt buffer containing 5 mM HEPES, 2 mM MgCl_2_, 0.1 mM EGTA and 2 mM DTT at pH 7.2 or assay buffer containing 20 mM HEPES, 40 mM NaCl, 4 mM MgCl_2_ at pH 7.3 for basal ATPase activity measurements with *Dd*MD.

### Cell culture maintenance

HeLa Kyoto H2B-mCherry eGFP-α-tubulin cells were purchased from Cell Lines Service GmbH and maintained in DMEM high glucose (4.5 g/L) supplemented with 10% FBS, 2 mM L-glutamine, 100 U/ml streptomycin, 100 μg/ml penicillin, 0.5 mg/ml G418 and 0.5 μg/ml puromycin. HeLa cells were a kind gift from Sára Tóth (Semmelweis University, Faculty of Medicine, Department of Genetics, Cell- and Immunobiology) and were maintained in low glucose DMEM (1 g/l) supplemented with 10% FBS, 2 mM L-glutamine, 100 U/ml streptomycin and 100 μg/ml penicillin. M2 cells ‒ a kind gift of Tom Stossel and Fumihiko Nakamura (Brigham & Women’s Hospital, Harvard Medical School, Translational Medicine Division) ‒ were grown in MEM (*Lonza*) supplemented with 8% newborn calf serum (*Sigma*) and 2% FCS. The cell cultures were maintained in a humidified atmosphere of 5% CO_2_ at 37 °C. Cells were grown as monolayers in T-75 cm^2^ culture flasks and subcultured by 0.02% EDTA (200 mg/l) 2–3 times each week when reached 90% confluency.

### Conditions of cellular assays

For blebbing index determination, M2 cells were incubated for 30 minutes in PBS in every case prior to the addition of inhibitors, in order to initiate extensive blebbing. Blebbing of the cells was monitored before as well as after inhibitor addition to the cells. DMSO concentration was 0.1 vol/vol% in all experiments. Blebbing index was calculated by summing the initiated blebs in a 5-minute interval (starting at the indicated time points) on a given standard area^17^ (whole cells with similar size, diameter = 12 ± 1 μm) and normalizing it to the blebbing index prior to inhibitor treatment.

For wound healing assays, 200 μl of HeLa cells were plated on 35 mm borosilicate glass imaging dishes at 300,000 cells/ml concentrations and incubated for 24 hours. 1 hour prior to wounding, cells were incubated in 20 μM of the different myosin II inhibitors. After one hour, scratches were made with pipette tips in monolayer cultures and the motility of cells for occupying the wound was monitored for 24 hours with a Foculus Digital Camera (IEEE1394). Analysis of cell motility was carried out by ImageJ, by measuring the distance between two lines fitted on the two sides of the approaching cell edges created by the pipette tips.

For cell growth assays, HeLa Kyoto H2B-mCherry eGFP-α-tubulin cells were grown on an eight-chambered Lab-Tek borosilicate cover glass system, with the initial cell concentration of 100,000 cells/ml. As soon as the cells attached to the surface, they were treated with 0, 2, 5, 10, 25 or 50 μM concentrations of para-amino-, para-nitro- or blebbistatin. Cells were grown for three days in the presence of the indicated inhibitor concentrations and cell numbers were quantified each day. The final vol/vol% of DMSO in all experiments was 0.1. Data analysis was carried out by Fiji software. On the third day of the experiment, multinuclear cells were visualized by using a Zeiss LSM 710 with a Plan Apo 20x/0.8 objective. For phototoxicity experiments, confocal time-lapse imaging of HeLa Kyoto H2B-mCherry eGFP-α-tubulin cells for 12 hours was performed in DMEM further supplemented with 25 mM HEPES in order to achieve CO_2_-independent media. Confocal time-lapse imaging was performed on a Zeiss LSM 710 using a Plan Apo 20x/0.8 objective, with the following parameters: 2% laser intensity at λ_exc_ = 488 nm, 3% laser intensity at λ_exc_ = 543 nm, 1.2× zoom, average 4, line step 1, 1000 × 1000 pixels, 0.35 μm pixel size, 0.65 μs dwell time, 3 slice/z-stack with 7 μm between slices at every 10 minutes.

### Fish husbandry and embryo treatments

Transgenic *Tg(−8.0cldnb:lynEGFP)*^*zf106*^ (*cldnb:EGFP*)^25^ zebrafish (*Danio rerio*) were maintained in the animal facility of Eötvös Loránd University. For the experiments investigating the skeletal and heart muscle of zebrafish, 6 dpf embryos were used. For cytotoxicity assays, 2 dpf embryos were dechorionated and placed in the indicated reagent concentrations. Embryos were kept in E3 medium (5 mM NaCl, 0.33 mM MgCl_2_, 0.33 mM CaCl_2_ and 0.17 mM KCl).

### Conditions of assays with zebrafish embryos

For the fast escape response experiments, 6 dpf embryos were placed in 50 μl drops of E3 medium on a plastic plate. They were treated with 0, 2, 5, 10 or 20 μM of the inhibitors. 10 taps were carried out with 1 minute intervals every hour for 4 hours. The tapping force was standardized. The videos were recorded by a Ximea Camera (MQ003MG-CM) at 500 fps for 2 s. Data were analyzed by Flote software version 2.1 and maximal angular velocity parameters of C-start reflex were quantified. For the experiments investigating the effect of the inhibitors on the heart muscle of zebrafish, 6 dpf larvae were embedded in 1% agarose and kept in E3 in the presence of 20 μM concentrations of the inhibitors. Videos were taken at 15.1 fps before and after inhibitor treatment every hour for 3 hours. For the cytotoxicity assay, zebrafish embryos were incubated in E3 medium containing 0 μM (Control), 5 μM, 10 μM or 20 μM of para-aminoblebbistatin, para-nitroblebbistatin or blebbistatin in 0.1 vol/vol% final concentration of DMSO and monitored for 70 hours. Embryos were considered to be dead when the whole body became necrotic and the heart stopped beating. Dead embryos were removed from the experiment to prevent contamination. Images of zebrafish were captured with a Zeiss Stereo Lumar V12 microscope using a NeoLumar S 0.8x FWD 80mm objective at 50× zoom.

All experiments were performed in accordance with relevant guidelines and regulations approved by the Hungarian National Food Chain Safety Office (Permit Number: XIV-I-001/515-4/2012).

## Additional Information

**How to cite this article**: Várkuti, B. H. *et al.* A highly soluble, non-phototoxic, non-fluorescent blebbistatin derivative. *Sci. Rep.* **6**, 26141; doi: 10.1038/srep26141 (2016).

## Supplementary Material

Supplementary Information

Supplementary Video 1

Supplementary Video 2

## Figures and Tables

**Table 1:** Inhibition of blebbing of M2 cells.

Concentration	5 μM #x003C4; (min)	10 μM τ (min)	25 μM τ (min)	50 μM τ (min)
Bleb	24.6 ± 2	10.5 ± 0.8	5.4 ± 1	–
NBleb	21.9 ± 4.6	11.7 ± 0.5	5.9 ± 0.2	–
AmBleb	25.0 ± 4.3	16.8 ± 1.6	9.9 ± 0.3	7.8 ± 0.3

Time constant (τ) ± s.d. values of inhibition of blebbing are determined from the fitted exponentials to data of Fig. 3b–d.
