# Supplementary material for: A highly soluble, non-phototoxic, non-fluorescent blebbistatin derivative
Source: Sci Rep. 2016 May 31;6:26141. doi: 10.1038/srep26141 (PMC4886532; doi:10.1038/srep26141)
Supplement: Supplementary Information [file srep26141-s1.pdf]

Supplementary Material

A highly soluble, non-phototoxic, non-fluorescent blebbistatin derivative

Boglárka H. Várkuti[1,+], Miklós Képiró[1,+], István Ádám Horváth[1], László Végner[1], Szilvia Ráti[1], Áron Zsigmond[2], György Hegyi[1], Zsolt Lenkei[3], Máté Varga[2] and András Málnási-Csizmadia[1,4,5,*]

[1]Department of Biochemistry, Institute of Biology, Eötvös Loránd University, H-1117 Budapest, Hungary

[2]Department of Genetics, Institute of Biology, Eötvös Loránd University, H-1117 Budapest, Hungary

[3]Brain Plasticity Unit, ESPCI-Paris-Tech, CNRS UMR8249, Paris, France

[4]MTA-ELTE Molecular Biophysics Research Group, Department of Biochemistry, Eötvös Loránd University, H-1117 Budapest, Hungary

[5]Optopharma Ltd., H-1015 Budapest, Hungary

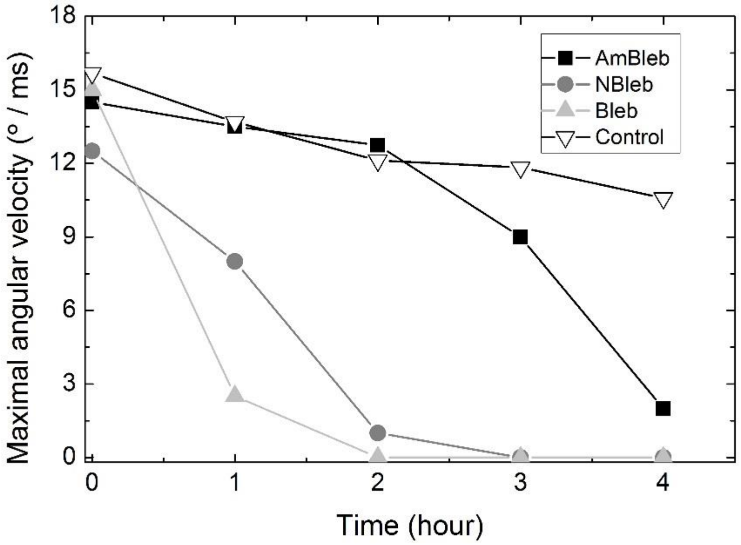

Supplementary Figure 1. Maximal angular velocity of the head during C-start escape of zebrafish in the absence and presence of blebbistatin (Bleb), para-nitroblebbistatin (NBleb) and para-aminoblebbistatin (AmBleb). Medians (n=30) of the parameter maximal angular velocity of C-start are presented in the function of time in the absence (Control) and in the presence of 2 µM concentration of the inhibitors. Half-inhibition values are 0.6, 1.3 and 3.2 hours for Bleb, NBleb and AmBleb, respectively.

Video Legends

Video 1. Fast escape response of fish in the absence or presence of blebbistatin (Bleb), para-nitroblebbistatin (NBleb) and para-aminoblebbistatin (AmBleb). Representative videos of the fast escape of single zebrafish embryos in drops due to a single tap on the experimental surface, after 1 hour of incubation in the absence (Control) or presence of 20 µM inhibitor concentrations. The strength of the tap was standardized by a custom-made tapping device. The fast escape response (maximal angular velocity of the fish) was monitored at 500 fps for 2 s.

Video 2. The effect of blebbistatin, para-nitroblebbistatin and para-aminoblebbistatin on the heart muscle of zebrafish embryos. Representative videos of the heart beating of the same zebrafish embryos before and after incubating the animals in the presence of 20 µM for 1 hour (Bleb, Nbleb) or for 3 hours (AmBleb). Videos were taken at 15 fps for 110 s.
